# The application of enhanced recovery after surgery (ERAS)/fast-track surgery in gastrectomy for gastric cancer: a systematic review and meta-analysis

**DOI:** 10.18632/oncotarget.18581

**Published:** 2017-06-20

**Authors:** Jie Ding, Benlong Sun, Peng Song, Song Liu, Hong Chen, Min Feng, Wenxian Guan

**Affiliations:** ^1^ Department of General Surgery, Nanjing Drum Tower Hospital, The Affiliated Hospital of Nanjing University Medical School, Nanjing, 210008 China

**Keywords:** enhanced recovery after surgery, fast-track surgery, gastric cancer, conventional care, meta-analysis

## Abstract

**Background:**

The study aimed to compare the safety and effectiveness of Enhanced recovery after surgery (ERAS) with conventional care in gastrectomy for gastric cancer.

**Methods:**

Search strategy from Pubmed, Embase, Web of science, Cochrane library and reference lists was performed. The collected studies were randomized controlled trials and published only in English, and undergoing ERAS in gastrectomy for gastric cancer from January 1994 to August 2016.

**Results:**

A total of eight studies including 801 patients were included. There were 399 cases in the ERAS and 402 cases in the conventional care groups. Meta-analysis showed that time to first passage of flatus (weighted mean difference (WMD) -14.57; 95% confidence interval (CI) -20.31 to -8.83, *p*<0.00001), level of C-reaction protein (WMD -19.46; 95 % CI -21.74 to -17.18, *p*<0.00001) and interleukin-6 (WMD-32.16; 95 % CI -33.86 to -30.46,*p*<0.00001) on postoperative days, postoperative hospital stay (WMD -1.85; 95 % CI -2.35 to -1.35, *p*<0.00001), hospital charge (WMD −0.94, 95 % CI, −1.40 to 0.49, *p*<0.0001) were significantly decreased for ERAS, but increased readmission rates (odds ratio (OR), 3.42, 95 % CI, 1.43 to 8.21, P=0.006). There were no statistically significant differences in intraoperative blood loss, operation time, number of retrieved lymph nodes, duration of foley catheter and postoperative complications (*p*>0.05).

**Conclusions:**

ERAS is considered to be safe and effective in gastrectomy for gastric cancer. Further larger, multicenter and randomized trials were needed to beresearched.

## INTRODUCTION

Gastric cancer is the fourth most common cancer and the third leading cause of cancer related deaths in the world [1], especially in Eastern Asia, Central and Eastern Europe, and South America [2]. In 2015, stomach cancer being the second most common type of cancer incident and the leading cause of cancer death in China [3]. At present, there had different interventional measures were applied in the perioperative period of gastric cancer which promotes patients recovery.

Enhanced recovery after surgery (ERAS), also known as fast track surgery (FTS), was initiated by Henrik Kehlet in 1990s [4, 5]. Over the past decade, the technique has been developed rapidly because of its significant benefits and safety [6]. ERAS is a multidisciplinary approach aiming to reduce the surgical stress response and organ dysfunction, therefore promoting patients postoperative recovery [6, 7]. The core components of ERAS include anesthesia and perioperative fiuid management, optimal pain control, early to eat and mobilization, among others (Table 1) [8]. In recent years, ERAS has been applied to different fields of surgeries, such as radical prostatectomy [9], cardiac surgery [10] and colorectal surgery [11-14]. In colorectal surgery, the ERAS working group developed [15] and modified the consensus guidelines for ERAS programs in 2009 [16]. These programs addressed 20 issues which included preadmission counseling, preoperative preparation, standard anesthetic protocol, postoperative care and so on.

**Table 1:** Characteristics of the studies included

Study	year	type of literature	Number		Age		Male/female		BMI		TNM(I/II/III/IV)		Approach	Outcome measures*
			FTS	CC	FTS	CC	FTS	CC	FTS	CC	FTS	CC		
Wang	2010	RCT	45	47	58.76±9.66	56.87±9.16	32/13	29/18	23.8±52.40	23.25±2.79	—	—	open	4, 6,7,8,9,10,11
Liu	2010	RCT	33	30	60.7±9.7	61.9±8.3	18/15	15/15	21.84±2.65	21.28 ± 2.54	—	—	open	4,5,6,7,8,10,11
Kim	2012	RCT	22	22	52.64±11.5	57.45±14.54	9/13	7/15	23.40±3.17	23.77±3.54	20/1/1/0	20/2/0/0	LAP	1,2,3,4,5,6,8,9,10,11
Hu(Open)	2012	RCT	21	20	59(49-71)	62.5(45-72)	9/12	12/8	23.54±2.59	23.47±2.62	1/8/11/1	1/6/11/2	open	1,2,3,4,6,8,9,10,11
Hu(LAP)	2012	RCT	19	22	64(40-71)	64.5(49-75)	10/9	10/12	22.94±2.23	22.99±2.24	1/10/8/0	1/10/10/1	LAP	1,2,3,4,6,8,9,10,11
Feng	2013	RCT	59	60	54.98±11.35	55.79±10.06	41/18	44/16	22.44±3.51	21.01±1.78	14/12/33/0	8/31/21/0	open	1,2,4,8,9,10,1
Bu A*	2015	RCT	64	64	62.4±7.8	63.0±7.4	31/33	35/29	21.3±1.7	21.8±2.2	9/34/21/0	13/32/19/0	open	1,2,4,8,10,11
Bu B*	2015	RCT	64	64	80.1±4.0	79.6±3.5	37/27	40/24	21.4±2.0	21.2±2.3	8/30/26/0	9/27/28	open	1,2,4,8,10,11
Abdikarim	2015	RCT	30	31	63 ± 12	62 ± 11	21/9	20/11	—	—	0/13/17/0	0/13/18/0	LAP	1,2,3,8,10,11
Liu(Open)	2016	RCT	21	21	67.8±3.9	68.6±4.9	9/12	11/10	22.0±1.9	21.4±1.8	3/9/9/0	3/10/8/0	open	1,2,4,6,7,8,9,11
Liu(LAP)	2016	RCT	21	21	69.2±5.1	70.3±5.8	10/11	12/9	21.5±2.0	21.9±2.3	2/10/9/0	1/9/11/0	LAP	1,2,4,6,7,8,9,11

Open = open gastrectomy; LAP = laparoscopic gastrectomy; A*=45-74 years old; B*=75-89 years old; BMI=body mass index (in kg/mm2); TNM = tumor, node and metastasis stage*1= blood loss, 2=operation time, 3=number of retrieved lymph nodes, 4=first passage of flatus, 5=duration of foley catheter, 6=C-reaction protein,7= interleukin-6; 8=postoperative hospital stay; 9= hospital charge, 10=readmission rate, 11=postoperative complications.

Reports on ERAS for gastric cancer are generally based on single study which would lack credibility, and also previously published results of meta-analysis were not comprehensive. So, we performed a meta-analysis to systematically describe the feasibility and safety of ERAS in patients undergoing gastrectomy for gastric cancer compared with conventional care. This meta-analysis was performed in line with the recommendations of the preferred reporting items for systematic reviews and meta-analyses (PRISMA) statement (Supplemental Table 1) [17].

## RESULTS

### Literature search

**Figure 1:**
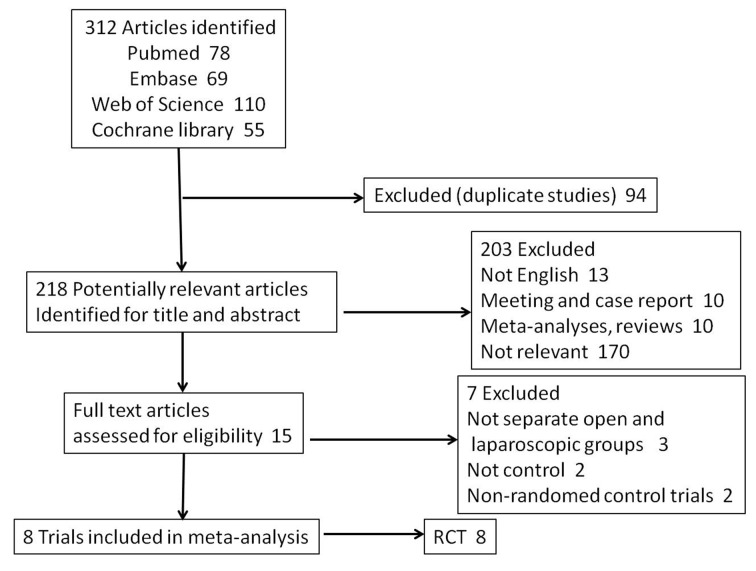
Selection process for studies included in the meta-analysis

Initially there were about 312 articles were searched from the above databases from January 1994 to August 2016. Based on the inclusion criteria, 297 studies were excluded, and 15 studies were subjected for a more detailed review. Three studies were excluded because there were no separate open and laparoscopic groups, 2 articles were eliminated because no set up control experiment, two studies were excluded due to non-RCTs. Finally, 8 studies [18-25] with a total of 801patients were included in this meta-analysis and all were single-center studies. Studies by Hu et al [18] and Liu et al [23] can be considered as two independent research studies in which they separately reported the effectiveness and safety of FTS for open and laparoscopic gastrectomy, when the results showed significant heterogeneity, a subgroup analysis was performed. The study by Bu et al [25] divided the patients into two groups: Bu (45-75 years old) and Bu (75-89 years old). In total, 8 articles were included for the meta-analysis (Figure 1). Characteristics of each trial are given in Table 1.There were 399 patients in the FTS group and 402 patients in the conventional care group.

### Methodological quality of included studies

Blinding is the most consistent risk of bias among randomized trials, since this type of surgical studies are not readily amenable to blinding, it was predictable that none of the trials were double-blinded. In addition to the studies by Hu et al [18] and Abdikarim et al, other studies had a score of four or more which showed a higher quality. Risk of bias assessment for randomized trials was shown in Table 2.

**Table 2:** Assessment of bias for included studies

	wang	Liu	Kim	Hu(Open)	Hu(LAP)	Feng	Bu A*	Bu B*	Abdikarim	Liu(Open)	Liu(LAP)
	2010	2010	2012	2012	2012	2013	2015	2015	2015	2016	2016
Random sequence generation (selection bias)	+	+	+	?	?	+	+	+	?	+	+
Allocation concealment (selection bias)	+	+	+	?	?	+	?	?	?	?	?
Blinding of participants and personnel (performance bias)	-	-	-	-	-	-	-	-	-	-	-
Blinding of outcome assessment (detection bias)	-	-	-	-	-	+	+	+	+	+	+
Incomplete outcome data (attrition bias)	+	+	-	+	+	+	+	+	?	+	+
Selective reporting (reporting bias)	+	+	+	+	+	+	+	+	+	+	+
Other bias	?	+	+	+	+	+	?	?	+	?	?
Score	4	5	4	3	3	6	4	4	3	4	4

+: risk of bias; -: high risk of bias; ?: unclear risk of bias.

### Number of FTS items

The number of FTS items in the four studies contained a mean of 11.3 (range 9 to 15) for 20 FTS items according to the guidelines of the ERAS working group [16]. The exact items used in each study were listed in Table 3.

**Table 3:** Number of ERAS items used in included studies

	wang	Liu	Kim	Hu(Open)	Hu(LAP)	Feng	Bu A*	Bu B*	Abdikarim	Liu(Open)	Liu(LAP)
	2010	2010	2012	2012	2012	2013	2015	2015	2015	2016	2016
Preadmission information and counseling	√	√	√	√	√	√	√	√	√	√	√
Preoperative bowel preparation	√	√	√	√	√		√	√	√	√	√
Preoperative fasting and preoperative carbohydrate loading	√	√	√	√	√	√	√	√	√	√	√
Preanesthetic medication	√										
Prophylaxis against thromboembolism											
Antimicrobial prophylaxis						√	√	√			
Standard anesthetic protocol	√						√	√	√		
Preventing and treating postoperative nausea and vomiting											
Laparoscopy-assisted surgery			√		√				√		√
Surgical incisions	√	√	√	√	√		√	√		√	√
Nasogastric intubation	√	√	√	√	√	√	√	√	√	√	√
Preventing intraoperative hypothermia	√	√	√			√	√	√			
Perioperative fluid management	√			√	√		√	√	√	√	√
Drainage of peritoneal cavity following colonic anastomosis	√			√	√	√					
Urinary drainage	√	√	√	√	√	√	√	√	√		
Prevention of postoperative ileus	√			√	√						
Postoperative analgesia	√		√			√	√	√	√	√	√
Postoperative nutritional care	√	√	√	√	√	√	√	√	√	√	√
Early mobilization	√	√	√	√	√	√	√	√	√	√	√
Audit											

### Effective outcome measures

#### Intraoperative blood loss

Intraoperative blood loss were reported in six studies [18, 21-25]. There was no significant difference observed in the intraoperative blood loss between ERAS and conventional groups (WMD -1.80; 95 % CI -7.71 to 4.12, *p* = 0.55, from fixed effects model). Also, no significant heterogeneity observed among the trials (I^2^ = 9%, *p* = 0.36) (Table 4).

**Table 4:** Meta-analysis of ERAS versus conventional care

Group	Observed outcomes	n^a^	WMD/SMD (95% CI)	*p*	Heterogeneity test	
					p^b^	I^2^(%)
ERAS/ conventional care	Intraoperative blood loss	9	-1.80(-7.71, 4.12)	0.55	0.36	9
	Operation time	9	-2.88(-6.21, 0.46)	0.09	0.69	0
	postoperative hospital stay	11	-1.85(-2.35, -1.35)	<.00001	<.00001	86
	Hospital charge	9	-0.94(-1.40, -0.48)	<.0001	<.00001	87

*P*=Test for overall effect, na =Number of comparisons, pb =*P* value of Q-test for heterogeneity test.

#### Operation time

Operation time was analyzed in six studies [18, 21-25]. There was no significant difference in the operation time between the ERAS and conventional groups (WMD -2.88; 95 % CI -6.21 to 0.46, *p* = 0.09, from fixed effects model), but operation time in patients undergoing FTS was less than those undergoing conventional care in the open group, which might be due to the completeness of the lymph node cleaning. Subgroup analysis revealed no significant heterogeneity among the trials (I^2^ = 0%, *p* = 0.69), neither in the open groups or laparoscopic groups. (Table 4)

#### Postoperative hospital stay

All included studies [18-25] reported postoperative hospital stay which was significantly lower for the ERAS group compared to the conventional perioperative care group (WMD -1.85; 95 % CI -2.35 to -1.35, *p* < 0.00001). There was significant heterogeneity observed among the trials (I^2^ = 86%, *p* < 0.00001), either in the laparoscopic (I^2^ = 70%, *p* = 0.02) or open groups (I^2^ = 88%, *p* < 0.00001) by subgroup analysis, indicating a random effects model. The postoperative hospital stay in patients undergoing ERAS was 2.03 days less than those undergoing conventional care in the open surgery group (WMD -2.03; 95% CI -2.73to -1.33, *p* < 0.00001), and in the laparoscopic group, the ERAS groups was 1.53 days less than that of the control group (WMD -1.53; 95 % CI -2.27 to -0.78, *p* < 0.0001), (Table 4).

#### Hospital charge

Hospital charge was reported in six articles [18, 19, 21-23, 25], which have different statistical units, so used SMD for analysis. The hospital charge were significantly less in the ERAS group than in the conventional group (SMD −0.94, 95 % CI, −1.40 to −0.48, *p* < 0.0001, from random effects model), with evidence of heterogeneity among the trials (I^2^ = 87 %, *p* < 0.00001), (Table 4).

#### Number of retrieved lymph nodes

**Figure 2:**
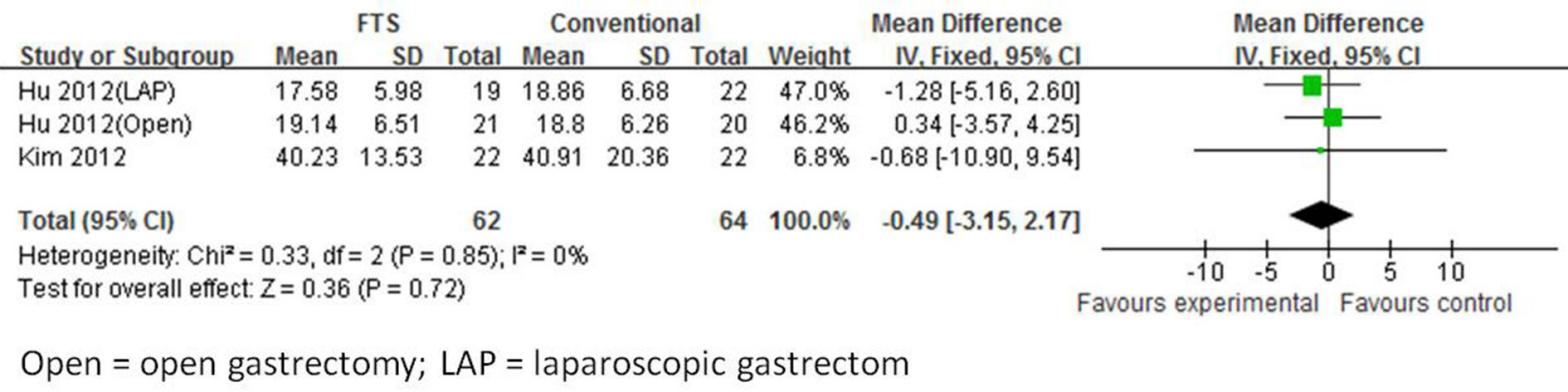
Forest plot of FTS *versus* conventional care for number of retrieved lymph nodes

Three studies [18, 22, 24] mentioned the number of retrieved lymph nodes. Abdikarim et al study was excluded because performing the total or distal subtotal gastrectomy, and another two studies included distal gastrectomy for gastric cancer. There was no significant difference observed in the number of retrieved lymph nodes between the ERAS and conventional groups (WMD -0.49; 95 % CI -3.15 to 2.17, *p* = 0.72, from fixed effects model). Also, no significant was observed heterogeneity among the trials (I^2^ = 0%, *p* = 0.85) (Figure 2).

#### First passage of flatus

**Figure 3:**
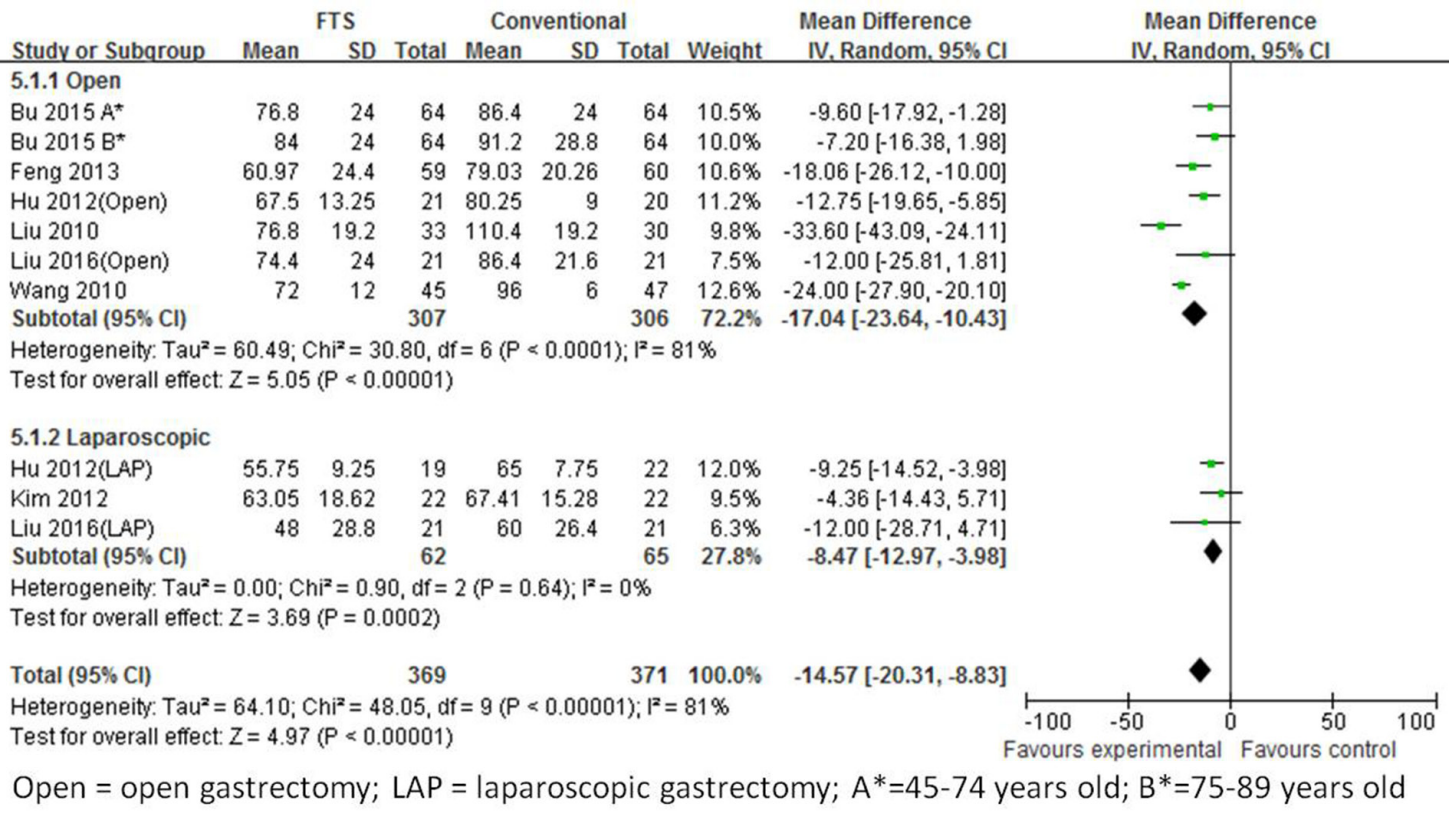
Forest plot of FTS *versus* conventional care for first passage of flatus

First passage of flatus was mentioned in seven [18-23, 25] studies. In addition to the laparoscopic groups (I^2^ = 0%, *p* = 0.64), other groups showed significant heterogeneity among the trials (I^2^ = 81%, *p* < 0.00001), indicating a random effects model. The first passage of the flatus with undergoing ERAS was 17.04 h less than those undergoing conventional care in the open surgery group (WMD -17.04; 95 % CI -23.64 to -10.43, *p* < 0.0001). However, in the laparoscopic group, the FTS group showed 8.47 h less than that of the control group (WMD -8.47; 95 % CI -12.97 to -3.98, *p* = 0.0002) (Figure 3).

#### Duration of foley catheter

**Figure 4:**
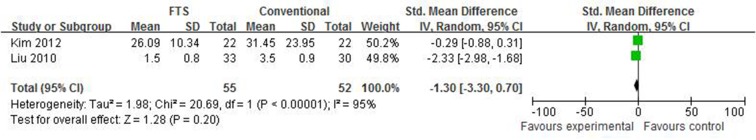
Forest plot of FTS *versus* conventional care for duration of foley catheter

Two studies [20, 22] recorded the duration of foley catheter and used different statistical units, so used SWD for analysis. There was no significant difference observed in the duration of foley catheter between the ERAS and conventional groups (SMD -1.30; 95 % CI -3.30 to 0.70, *p* = 0.20, from random effects model). Significant heterogeneity was observed among the trials (I^2^ = 95%, *p* < 0.00001) (Figure 4).

#### C-reaction protein

It has been established that a higher rate of serious postoperative complications was associated with an extreme response to surgical stress [26], and that the CRP and interleukin (IL)-6 may act as markers for the severity of surgical stress response [27, 28]

**Figure 5:**
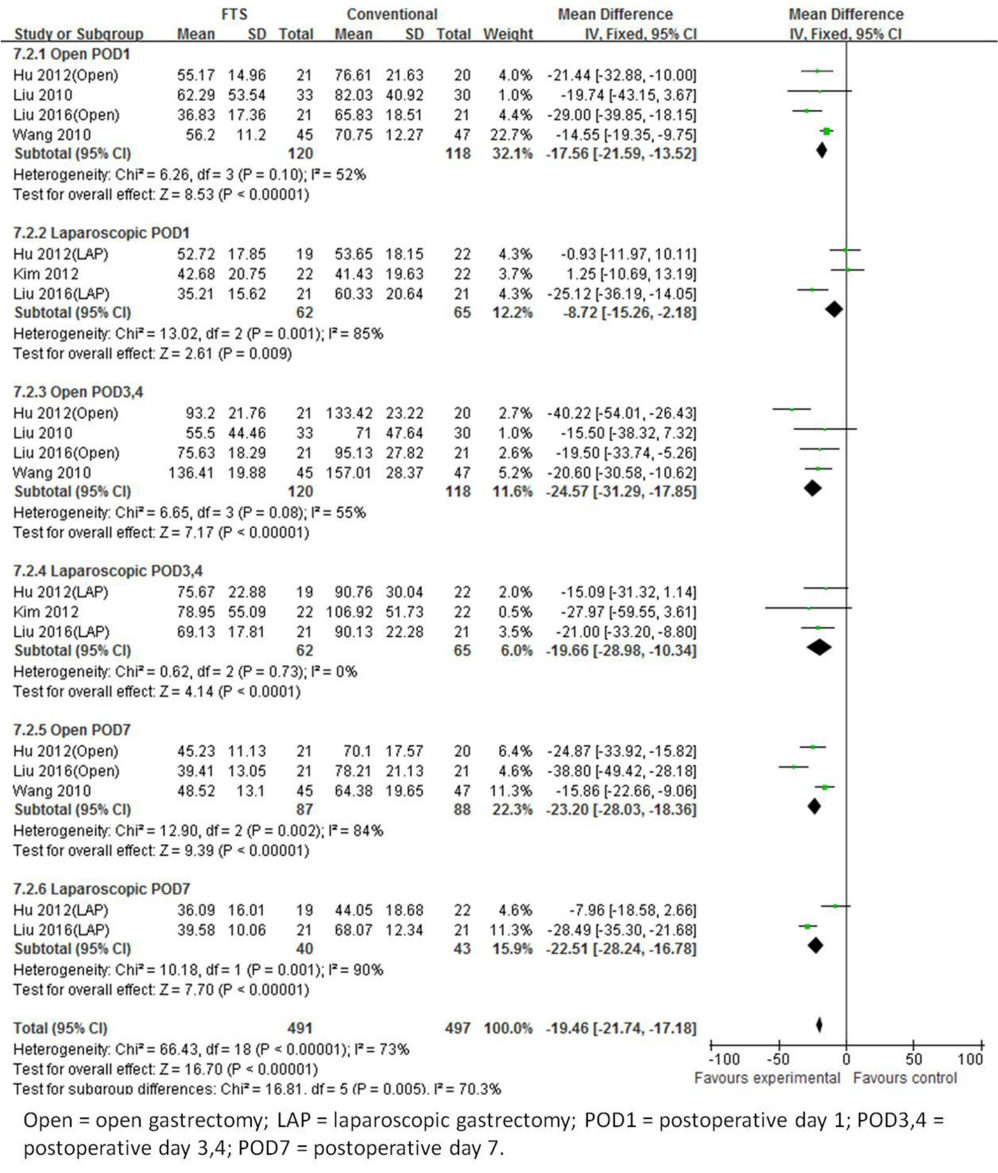
Forest plot of FTS *versus* conventional care for C-reactive protien

There are five studies [18-20 22, 23] that recorded CRP levels on postoperative days (POD) 1, 3 or 4 and 7. Heterogeneity among the trials was significant (I^2^ = 73%, *p* < 0.00001), indicating a random effects model. All subgroup analyses indicated a decreased CRP levels in the ERAS group patients. Overall, the CRP level of patients undergoing FTS was 19.46 mg/L which was less than those patients undergoing conventional care in the open surgery group (WMD -19.46; 95 % CI -21.74 to -17.18, *p* < 0.00001) (Figure 5).

#### Interleukin-6

**Figure 6:**
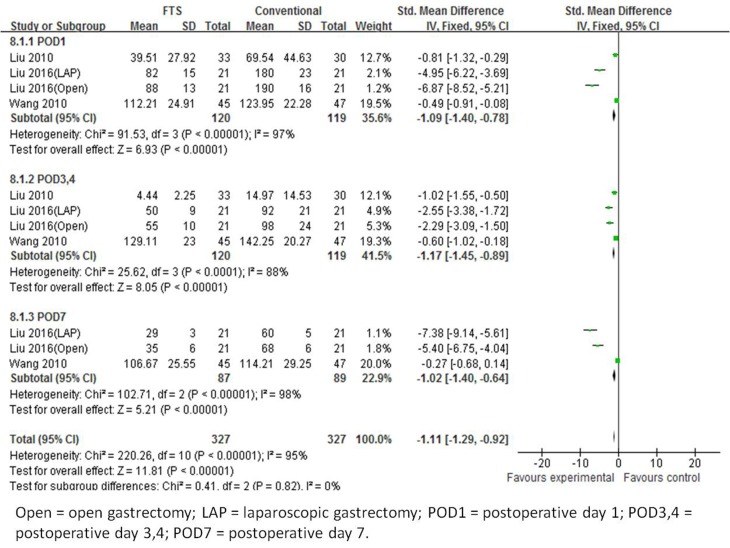
Forest plot of FTS *versus* conventional care for interleukin-6

Three articles [19, 20, 23] studied the IL-6 levels on POD 1, 3 or 4 and 7. There were obvious differences observed among them in the levels of IL-6, and we used SMD for analysis. The heterogeneity among the trials was observed to be significant (I^2^ = 95%, *p* < 0.00001), indicating a random effects model. The IL-6 levels of patients undergoing ERAS were less than those patients undergoing conventional care on POD 1, 3 or 4 and 7 (Figure 6).

### Safety outcome measures

#### Readmission rate

**Figure 7:**
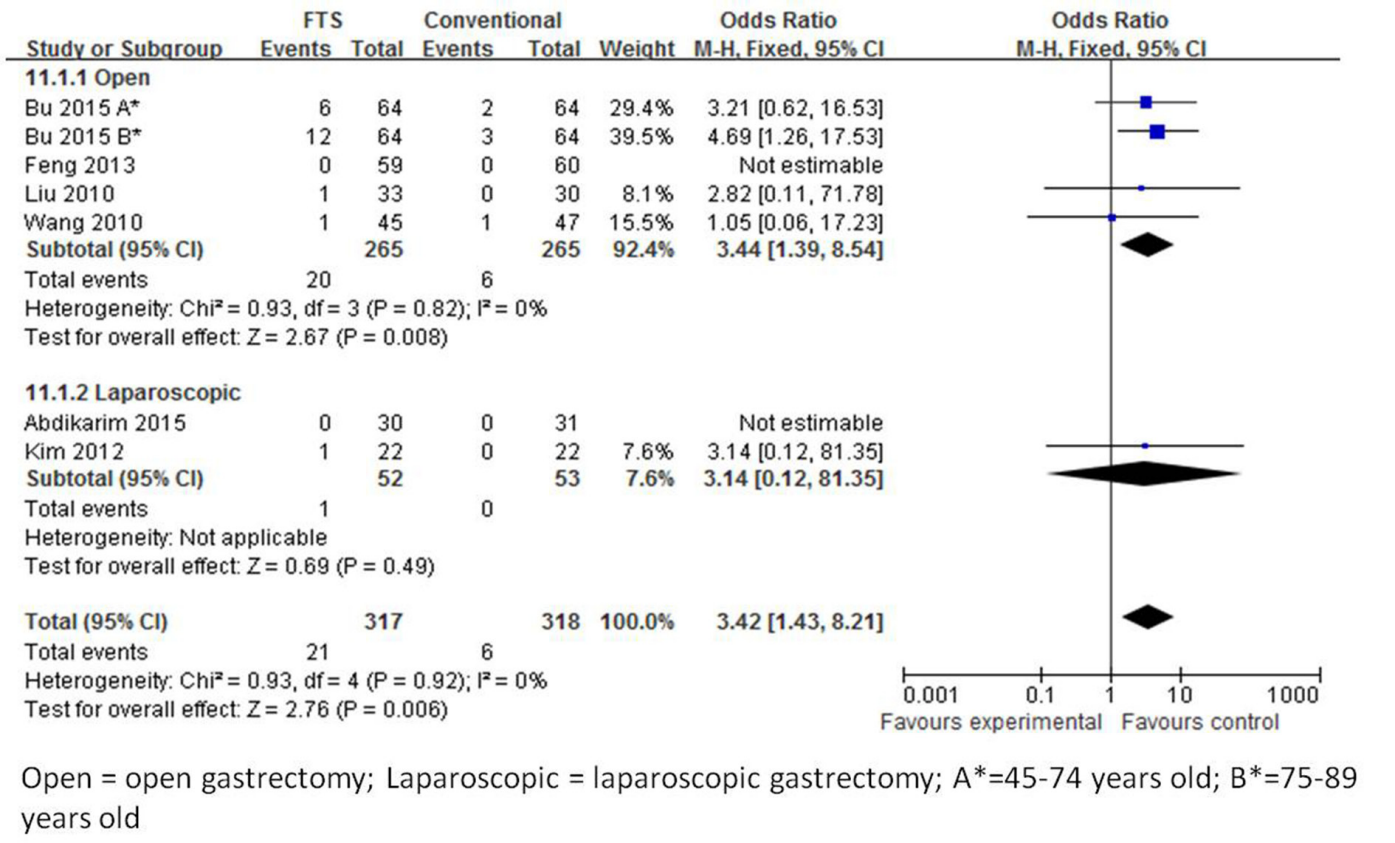
Forest plot of FTS *versus* conventional care for readmission rates

Readmission rates were reported in six studies [19-22, 24, 25]. The readmission rates were significantly high in the ERAS group than in the conventional group (OR, 3.42, 95 % CI, 1.43 to 8.21, *p* = 0.006, from fixed effects model), with no heterogeneity observed among the trials (I^2^ = 0 %, *p* = 0.92), (Figure 7).

#### Postoperative complications

**Figure 8:**
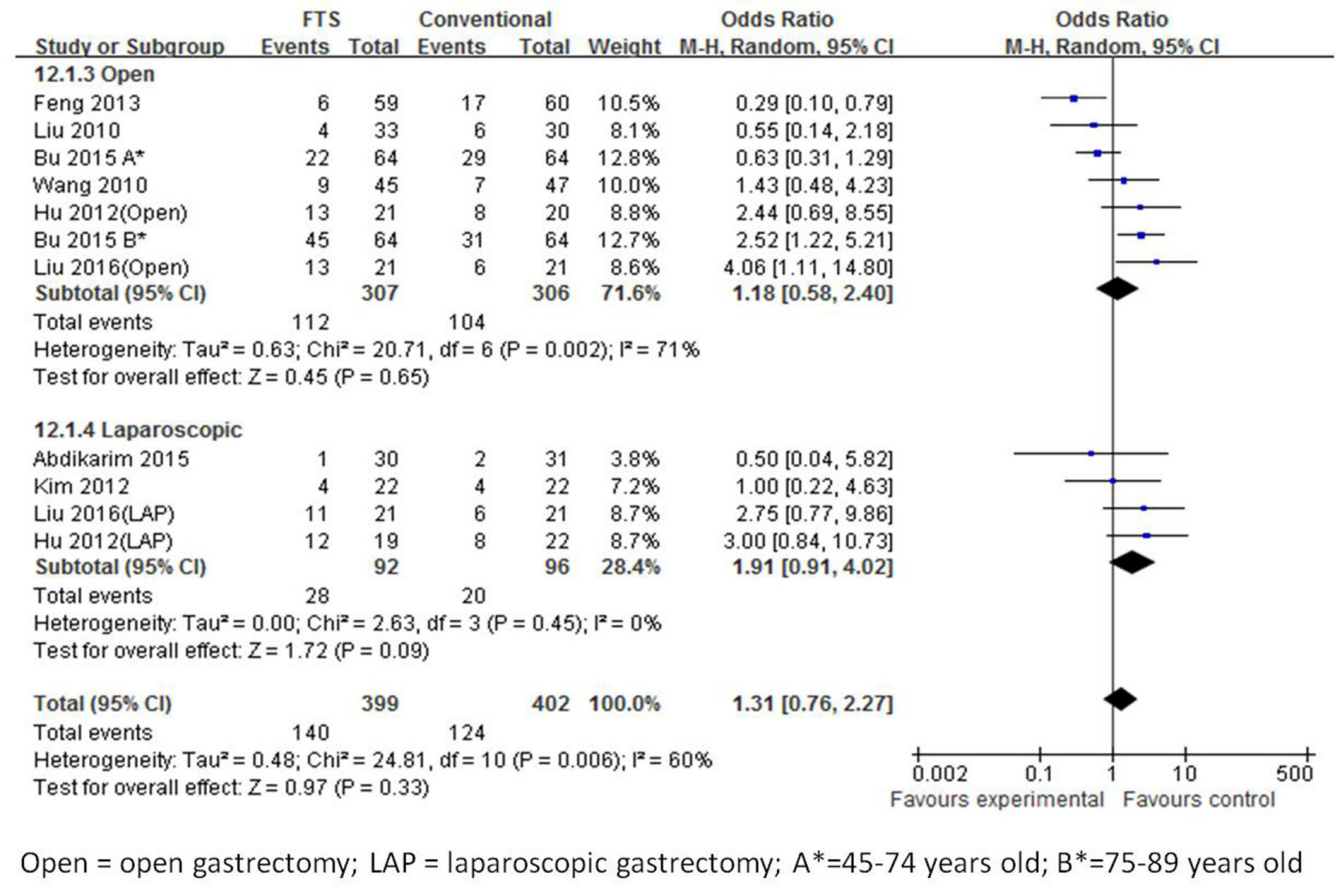
Forest plot of FTS versus conventional care for postoperative complication

All eight studies [18-25] calculated the postoperative complications. There was a significant heterogeneity observed among the trials in the open surgery group (I^2^ = 71 %, *p* = 0.002), but not in the laparoscopic group. In the random effects model, there was no significant difference observed in the postoperative complications between the ERAS and conventional perioperative care groups (OR 1.31; 95 % CI 0.76 to 2.27, *p* = 0.33), similarly between the open and laparoscopic groups (Figure 8).

### Publication bias

**Figure 9:**
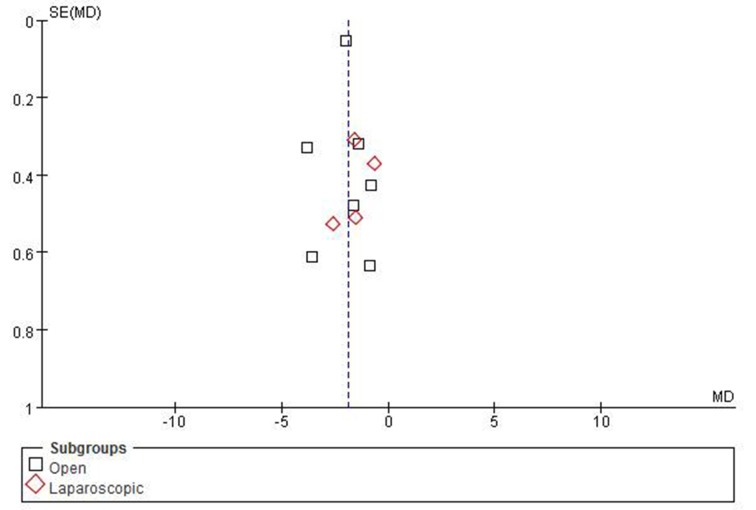
Funnel plots of publication bias

Publication bias may be present when there were fewer than 10 studies in the meta-analysis because the lower number of studies implies inherent weaknesses in the review. Funnel plots for postoperative hospital stay including all studies as follow in Figure 9, which showed publication bias has little effect for this meta-analysis.

## DISCUSSION

Our study aimed to evaluate the safety and effectiveness of ERAS in gastric cancer patients undergoing gastrectomy compared to the conventional care. Meta-analysis showed that ERAS have a significant reduction in time to first passage of flatus, postoperative CRP and IL-6, postoperative hospital stay, hospital charge compared with conventional care. We found no significant differences in intraoperative blood loss, operation time, number of retrieved lymph nodes, duration of foley catheter and postoperative complications between the groups. In addition, the readmission rates were significantly higher in the ERAS groups than conventional care.

Previously, some studies have been confirmed that ERAS have advantage compared with conventional care, but there are exist defects, such as outcome measures less and sample size insufficient, etc. Beamish et al published a systematic review and meta-analysis [29]that include eight RCTs. Among of them, five were English studies, two Chinese studies and one Japanese study. They concluded that postoperative hospital stay was significantly decrease in ERAS when compared to the control group patients. Also a reduced serum infiammatory responses and a lower cost were observed [29]. However our meta-analysis included eight RCTswhich were published only in English and were from Asia, which might be due to the higher incidence rates of gastric cancer in that region. The present work included three RCTs published recently in two years, which pointed out that ERAS was safe and effective compared to the conventional care in gastric cancer.

In the study of Chen S et al [30], among of 7 RCTs, there have two studies [31,32] from Chinese Journal of Clinical Nutrition, they showed that ERAS could shorter postoperative hospitalization and less hospitalisation expenditure. In addition to the above, our meta-analysis shown that ERAS also can promote the intestinal function recovery and reduce the postoperative stress reaction. Wang LH et al [33] collected 24 RCTs, but only 2 studies on gastric cancer research, and Chen ZX et al [34] only included 3 RCTs, it is obvious that they included study were fewer. Li YJ et al [35] and Yu Z et al [36] included 5 RCTs respectively, but they did not contain 3 newest RCT studies [23-25]. These authors included literatures are fewer, and at the same time the research mainly concentrated in the 2012 and 2010. In recent years, with ERAS develop rapidly around the world, especially in China, Japan and Korea, we urgently need a strong evidence of evidence-based medicine to support and confirm the advantage of ERAS in gastric cancer surgery, so we made a detailed search strategy, finally include 8 RCTs, which contains three newest papers [23-25], we could fully evaluate the advantage of ERAS compared with conventional care, than provide important medical evidence for the implementation of the ERAS.

The meta-analysis results revealed that ERAS can obviously reduce time to first passage of flatus compared to conventional care by the overall analysis or the subgroup analysis (the open and laparoscopic surgery subgroups), which contributed to promote intestinal function recovery and restore early enteral nutrition. Studies have shown that during fasting, the peristalsis of stomach and small intestine is slow and irregular contraction waves, while eating peristalsis is frequent and regular contraction waves [20]. So, early postoperative eating can accelerate the recovery of intestinal function [37], in addition, early enteral nutrition not only provide nutrients needed for the recovery of the body, also maintains the intestinal mucosal barrier function, reduces infection, thus accelerating organ recovery [38].

One of the major concerns about ERAS is higher risk associated with postoperative complications. In our study, the results showed that ERAS did not increase the possibility of postoperative complications compared to the conventional care by the overall analysis or the subgroup analysis. However, ERAS groups had high readmission rates than conventional care, as well as result in the open group, which might be due to that ERAS groups had significant shorter hospitalization time and some complications which did not happen or discover during the stay in hospital, such as intestinal obstruction, deep incision infection, even anastomotic leakage.

A high risk of statistical heterogeneity was identified among the 8 RCTs for the first passage of flatus, duration of foley catheter, CRP, IL-6, postoperative hospital stay, hospital charge and postoperative complications. In addition to statistical heterogeneity, there was some clinical heterogeneity exist, such as surgeon skill, the definition of inclusion and exclusion criteria were not consistent, selective differential treatments from the ERAS items among the studies and so on. These items may potentially weaken the findings of the present analysis. The heterogeneity of methodology cannot be completely ignored as bias risk and different study designs may affect the results of our analysis. So, we need to design a more reasonable and scientific study to reduce heterogeneity in the future.

ERAS protocols comprises of a new and revolutionary perioperative treatment that has become prevalent in gastric cancer [39], rectal cancer [40], head and neck oncology [41], Bariatric Surgery [42] and so on. In the last few years, many ERAS protocols have been suggested by hospital groups that include different fast track elements such as preoperative counseling and feeding, no bowel preparation, active prevention of hypothermia, and no routine use of nasogastric tubes and drains [43,44]. Overall, our findings indicated that effective analgesia can promote early mobilization, decrease the hospital stay and hospital charge, effective management during perioperative period could reduce the stress response and organ dysfunction, obviously promote the full recovery, than improve the hospital bed utilization rate and reduce the financial burden for patient’s family.

There were some limitations to this meta-analysis. Firstly, the present study contained only RCTs. Secondly, three studies were not blinded, so observer bias may exist. Thirdly, ERAS program depends on a well-trained and experienced multidisciplinary team which often include anesthesiologists, surgeons, dieticians and professional nursing staff, but this study involved different clinical centers and surgeons. Therefore, the operation time, intraoperative blood loss, postoperative hospital stay and hospital charge might be have some differences. Fourthly, there were some clinical and statistical heterogeneity between the included studies. All the included studies were done in East Asia, so the results might not be generalized to the Western countries. Unpublished studies and data might exist which influence our results. Finally, few studies and small number of patients were included, so the results cannot be generalized to a larger group and further studies are needed.

In conclusion, our results showed that ERAS program could shorten the time of fiatus, accelerate the decrease in CRP and IL-6 levels, shorten the postoperative hospital stay, and reduce the hospital charge. Thus, ERAS was effective and safe in some aspects—for gastric cancer patients who underwent open or laparoscopic gastrectomy surgery. However, due to the small number of studies available and their underlying heterogeneity, further multicenter and randomized control trials are required to study.

## MATERIALS AND METHODS

### Retrieval strategies

Two authors (J.D and B.L.S) independently performed a bibliographic search in Pubmed, Embase, Web of science and Cochrane library from January 1994 to August 2016, and only English literatures were collected. Review articles were also identified to find any other additional eligible studies. Research words included enhanced recovery after surgery, ERAS, fast track surgery, FTS, gastric cancer, gastrectomy.

### Inclusion and exclusion criteria

The inclusion criteria included were (1) evaluation of ERAS or FTS in comparison to the conventional care, (2) randomized-controlled trials (RCTs), (3) diagnosis of gastric cancer based on clinical symptoms, imaging, and pathology, (4) only English literature, and (5) the statistical outcomes were independent in open groups and laparoscopic groups.

Studies were excluded if they (1) reported fewer than four outcome measures as mentioned below: intraoperative blood loss, operation time, number of retrieved lymph nodes, first passage of flatus, duration of foley catheter, C-reactive protein, interleukin-6, postoperative hospital stay, hospital charge, readmission rate and postoperative complications, (2) were review, retrospective studies, case reports and unpublished studies with only abstracts presented at the national and international meetings, (3) lack or unable to extract the data, and (4) included gastric benign disease undergoing gastrectomy.

### Literature assessment and data extraction

Two reviewers (P.S and H.C) independently evaluated the quality of each eligible study according to the risk of bias tables from the Cochrane Handbook [45], across domains of selection, performance, detection, attrition, reporting, and other possible bias. Studies achieving a score of four or more from a maximum of seven were considered to be of higher quality.

Data was extracted from all the included articles independently by two authors (P.S and H.C) and In case of any discrepancy, we sorted out either by discussion or by a deciding arbiter (S.L). From each eligible study, the following information was extracted: first author, year of publication, number of patients, age, sex, body mass index (BMI), tumor, node, metastasis (TNM) stage, and surgical procedures of both cases and controls; number of ERAS items used according to the guidelines of the ERAS working group [16]. Eleven outcome variables were considered suitable for analyzing the effectiveness of ERAS: Intraoperative blood loss, operation time, number of retrieved lymph nodes, first passage of flatus, duration of foley catheter, C-reactive protein, interleukin-6, postoperative hospital stay, hospital charge, readmission rate and postoperative complications.

#### Statistical analysis

Statistical analyses were performed using RevMan version 5.0 (Nordic Cochrane Centre, Copenhagen, Denmark). When the statistics were unit consistent, weighted mean differences (WMD) were used, and in case of inconsistency or have obvious mean difference, standardized mean difference (SMD) was used. WMD or SMD with 95% confidence intervals (CIs) were calculated for continuous variables (blood loss, operation time, number of retrieved lymph nodes, first passage of flatus, duration of foley catheter, C-reactive protein, interleukin-6, postoperative hospital stay and hospital charge) by a fixed effects model or random effects model according to the heterogeneity assumption. The Q test was used to assess the presence of heterogeneity and the *I^2^*index to quantify the extent of heterogeneity [46], a random effects model was used to pool the studies by *p* of Q test ≤ 0.1 and *I*^*2 >*^ > 50% which indicated significant heterogeneity, otherwise, a fixed effects model was used. Odds ratios (ORs) and 95 % CIs were used to analyze the readmission rates and postoperative complications. If the study provided medians and interquartile ranges instead of means and standard deviations (SDs), we imputed the means and SDs as described by Hozoet al [47]. For all comparisons, except those for heterogeneity, statistical significance was defined as *p* < 0.05. Once overall results of analysis had significant heterogeneity, next a subgroup analysis was performed for open and laparoscopic groups as conditions. Funnel plots were synthesized to determine the presence of publication bias.

## SUPPLEMENTARY MATERIALS TABLE


